# De novo variants in *CACNA1E* found in patients with intellectual disability, developmental regression and social cognition deficit but no seizures

**DOI:** 10.1186/s13229-021-00473-3

**Published:** 2021-10-26

**Authors:** Beryl Royer-Bertrand, Marine Jequier Gygax, Katarina Cisarova, Jill A. Rosenfeld, Jennifer A. Bassetti, Oana Moldovan, Emily O’Heir, Lindsay C. Burrage, Jake Allen, Lisa T. Emrick, Emma Eastman, Camille Kumps, Safdar Abbas, Geraldine Van Winckel, Undiagnosed Diseases Network, Nadia Chabane, Elaine H. Zackai, Sebastien Lebon, Beth Keena, Elizabeth J. Bhoj, Muhammad Umair, Dong Li, Kirsten A. Donald, Andrea Superti-Furga

**Affiliations:** 1 Division of Genetic Medicine, Lausanne University Hospital (CHUV) and University of Lausanne, Lausanne, Switzerland; 2 Division of Autistic Spectrum Disorders, Department of Psychiatry, Lausanne University Hospital (CHUV), Lausanne, Switzerland; 3 Department of Molecular and Human Genetics, Baylor College of Medicine, Houston, TX USA; 4 Division of Medical Genetics, Department of Pediatrics, Weill Cornell Medicine, New York, NY USA; 5 Serviço de Genética Médica, Departamento de Pediatria, Hospital de Santa Maria, Centro Hospitalar Universitário de Lisboa Norte, Lisbon, Portugal; 6 Center for Mendelian Genomics and Program in Medical and Population Genetics, Broad Institute of MIT and Harvard, Cambridge, MA USA; 7 The Broad Institute of MIT and Harvard, Cambridge, MA USA; 8 Department of Neurology, Baylor College of Medicine, Houston, TX USA; 9 Department of Pediatrics, Baylor College of Medicine, Houston, TX USA; 10 Department of Paediatrics and Child Health, Faculty of Health Sciences, University of Cape Town, Cape Town, South Africa; 11 Department of Biochemistry, Faculty of Biological Sciences, Quaid-i-Azam University, Islamabad, Pakistan; 12 Division of Human Genetics, Department of Pediatrics, The Children’s Hospital of Philadelphia, Philadelphia, PA USA; 13 Department of Pediatrics, Perelman School of Medicine at the University of Pennsylvania, Philadelphia, PA USA; 14 Unit of Paediatric Neurology and Pediatric Neurorehabiliation, Woman-Mother-Child Department, Lausanne University Hospital, Lausanne, Switzerland; 15 Center for Applied Genomics, The Children’s Hospital of Philadelphia, Philadelphia, PA USA; 16 Medical Genomics Research Department, King Abdullah International Medical Research Center (KAIMRC), King Saud Bin Abdulaziz University for Health Sciences, Ministry of National Guard Health Affairs, Riyadh, Saudi Arabia; 17 Department of Life Sciences, School of Science, University of Management and Technology (UMT), Lahore, Pakistan; 18 Department of Paediatrics and Child Health, Red Cross War Memorial Children’s Hospital, Cape Town, South Africa; 19 Neuroscience Institute, Faculty of Health Sciences, University of Cape Town, Cape Town, South Africa

**Keywords:** Autism spectrum disorder, *CACNA1E*, Developmental regression, Epilepsy, Exome sequencing, Global developmental delay, Intellectual disability, Neurodevelopmental disorders, Seizures, Topiramate

## Abstract

**Background:**

De novo variants in the voltage-gated calcium channel subunit α1 E gene (*CACNA1E*) have been described as causative of epileptic encephalopathy with contractures, macrocephaly and dyskinesias.

**Methods:**

Following the observation of an index patient with developmental delay and autism spectrum disorder (ASD) without seizures who had a de novo deleterious *CACNA1E* variant, we screened GeneMatcher for other individuals with *CACNA1E* variants and neurodevelopmental phenotypes without epilepsy. The spectrum of pathogenic *CACNA1E* variants was compared to the mutational landscape of variants in the gnomAD control population database.

**Results:**

We identified seven unrelated individuals with intellectual disability, developmental regression and ASD-like behavioral profile, and notably without epilepsy, who had de novo heterozygous putatively pathogenic variants in *CACNA1E*. Age of onset of clinical manifestation, presence or absence of regression and degree of severity were variable, and no clear-cut genotype–phenotype association could be recognized. The analysis of disease-associated variants and their comparison to benign variants from the control population allowed for the identification of regions in the CACNA1E protein that seem to be intolerant to substitutions and thus more likely to harbor pathogenic variants. As in a few reported cases with *CACNA1E* variants and epilepsy, one patient showed a positive clinical behavioral response to topiramate, a specific calcium channel modulator.

**Limitations:**

The significance of our study is limited by the absence of functional experiments of the effect of identified variants, the small sample size and the lack of systematic ASD assessment in all participants. Moreover, topiramate was given to one patient only and for a short period of time.

**Conclusions:**

Our results indicate that *CACNA1E* variants may result in neurodevelopmental disorders without epilepsy and expand the mutational and phenotypic spectrum of this gene. *CACNA1E* deserves to be included in gene panels for non-specific developmental disorders, including ASD, and not limited to patients with seizures, to improve diagnostic recognition and explore the possible efficacy of topiramate.

**Supplementary Information:**

The online version contains supplementary material available at 10.1186/s13229-021-00473-3.

## Background

Voltage-gated calcium channels (VGCCs) are proteins that regulate the entry of calcium ions (Ca^2+^) in response to depolarization of the cellular membrane of excitable cells [1] and represent principal gateways for Ca^2+^ in nerve and both skeletal and cardiac muscle cells [2]. VGCCs are constituted by 5 different subunits, α1, α2, β, δ, γ, which together form the high-voltage-activated Ca^2+^ channels [3]. The α1 subunit is the main subunit indispensable for the functioning of the channel. It is composed of four homologous domains (I–IV), each containing 6 transmembrane segments, including a voltage sensor segment (S4) and 2 segments forming a pore (S5–S6) [1]. In mammals, ten α1 subunits have been described, encoded by ten different genes. Each of these subunits has specific molecular, genetic, physiologic and pharmacological properties [1, 4], and pathogenic variants in these genes have been associated with various human diseases and represent putative targets for treatment [5].

The α1E gene (*CACNA1E*) encodes the high-voltage-activated Cav2.3 type R calcium channel, which is expressed in various areas of the central nervous system, including the cerebellum [1]. More specifically, it is involved in the initiation of the presynaptic calcium entry and post-synaptic transmitter release [6].

*CACNA1E* variants were initially associated with seizures (Baker JJ 2016: http://epostersonline.com/acmg2016/node/2302) and with autism spectrum disorder (ASD) [7]. Other studies found a possible impact of *CACNA1E* single nucleotide polymorphisms in different human diseases and disorders including diabetes [8], migraine [9] and pain regulation [10]. More recently, the pathogenic role of *CACNA1E* variants in human epilepsy and neurodevelopmental disorders (NDD) has been described. One large genomic study of trios presenting with NDD identified *CACNA1E* as a candidate gene for NDD with or without epilepsy [11], and subsequently, variants in *CACNA1E* have been found in patients with developmental and epileptic encephalopathy (DEE) [12]. A large multiplex gene network analysis identified *CACNA1E* as candidate gene for epilepsy and autism [13]. Subsequently, a genetic association between calcium channels in general and ASD was further suggested by Liao and Li [14] and Graziano et al. [15].

The largest cohort of *CACNA1E* patients, described by Helbig et al. [12], reports 30 patients, aged between 1 and 16 years, who presented with severe to profound developmental delay and a combination of epilepsy, tone disorders and/or abnormal movements. Among these individuals, 88% were described as non-verbal and non-ambulatory. Of the 30 patients, 29 patients presented with hypotonia, six presented with spastic quadriplegia, 13 with joint contractures, 14 with extrapyramidal signs, and 26 with epilepsy (among these, 14 had epileptic spasms). Nine patients presented with developmental regression associated with the onset of epilepsy [12]. Interestingly, topiramate was the main anti-seizure drug reported to show a reduction in seizure presentation. Functional analysis of the Cav2.3 channel in 14 patients revealed a gain-of-function effect of the *CACNA1E* pathogenic variants with either a facilitated activation or an increased Ca^2+^ current. As topiramate blocks R-type calcium channels, among its different actions, the R-type calcium current might represent a common neuropathogenic mechanism involved in epilepsy and developmental delay [16].

We identified a de novo missense variant in *CACNA1E* in a patient with global developmental delay (GDD), ASD and successive developmental regressions unrelated to epileptic seizures. We used GeneMatcher [17] to seek other similar developmental patterns associated with *CACNA1E* variants, with the aim to identify possible neurodevelopmental presentations without epilepsy and to extend the phenotypic description of *CACNA1E*-related disorders. Using large datasets of control populations, we tried to compute the mutational constraint within *CACNA1E* domains and segments to identify putative functionally relevant segments that are more likely to harbor pathogenic variants.

## Method

### Participants

A missense de novo variant in *CACNA1E* was found in our index patient after analysis of an extended genetic panel, including epilepsy and developmental delay related genes. His phenotype was notable for two episodes of acute regression, in the context of a pre-existing GDD and ASD. Notably, he never presented with any type of epileptic seizures, the common feature in the cohort described by Helbig et al*.* [12]. Through GeneMatcher, we identified six additional patients who were carriers of heterozygous de novo putative deleterious variants in *CACNA1E* and presented with GDD, developmental regression and no epilepsy. Except for one patient aged 2, five patients presented with behavioral disturbances belonging to the field of ASD, despite the lack of precise behavioral phenotyping required for an ASD diagnosis. None of these patients has been reported previously.

### Sequencing and filtering of variants

Genomic DNA was extracted from whole blood from the patients and their parents. Exome sequencing in trios was done for 5/7 patients, and in the proband only for 2/7 patients. Segregation of the *CACNA1E* variants was done by Sanger sequencing for 6/7 patients. The protocols and software versions used for exome capture, sequencing, next-generation sequencing (NGS) pipeline and analysis for each patient are available in Additional file 1: Table S1. All discovered variants are defined in GRCh37 and the NM_000721.4 isoform of *CACNA1E*, and were classified following guidelines from the American College of Medical Genetics (ACMG) [18].

### Analysis of regional intolerance to missense variants in *CACNA1E*

We have downloaded the table of Missense Tolerance Ratio (MTR) scores for *CACNA1E* from MTR-Viewer v2 [19]. MTR is the measure of the proportion of the observed and expected missense variations in a gene given the specific protein regions, reflecting selective constraints of the given region. An MTR score above 1 suggests a positive selection of the segment, MTR score equal to 1 indicates segments’ selective neutrality, and an MTR value below 1 indicates purifying selection against missense alterations. A false discovery rate (FDR) value of < 0.1 is used to determine whether MTR deviates significantly from 1.0, to exclude false positives.

We have calculated the mean MTR score and the mean FDR for each CACNA1E segment, intersegment and interdomain regions. We computed the median, 25th percentile and 5th percentile MTR values (0.79, 0.60 and 0.33, respectively). We considered regions being selected against missense variants if their MTR value was below 1 with the associated FDR below 0.1, and being highly intolerant to missense alterations if their MTR value was below the 5th percentile with the respective mean FDR below 0.1, as previously described [20].

## Results

### Clinical reports

The seven patients, including the index patient and 6 patients recruited via GeneMatcher [17], were all bearing hitherto undescribed de novo heterozygous variants in *CACNA1E* (see Table 1). Shared clinical features among these patients were GDD, marked speech and language delays and social behavioral deficit. Furthermore, muscular hypotonia, motor stereotypies and sensory issues (a clinical criteria for ASD) were each reported in 5 of 7 patients. Four patients (4/7, 57%) also presented with developmental regression, unrelated to any seizures or specific electro-encephalographic (EEG) epileptic activity. The loss of previously acquired vocabulary and of communicative skills seen in these patients fulfilled the diagnostic criteria for so-called autistic regression. One adult patient (Patient 4) had a history of epileptic spasms, successfully treated with adrenocorticotropic hormone, without subsequent seizures in childhood or adulthood. No recognizable facial phenotype was identified in the patients. None of the patients had epilepsy, spastic quadriplegia, joint contractures, extrapyramidal signs or macrocephaly, as described in Helbig et al. [12]. An overview of the major clinical features seen in our patients is described in Table 1, and a more complete list for each case is available in Additional File 1: Table S2.

**Table 1:** Overlapping phenotypes in the *CACNA1E* patients

	Patient 1	Patient 2	Patient 3	Patient 4	Patient 5	Patient 6	Patient 7
*Genetics*							
*CACNA1E* variant (NM_000721.4) Coordinates in hg19	c.488T>C (p.Met163Thr) 1:181480622T>C	c.1499A>G (p.Gln500Arg) 1:181686412 A>G	c.2060C>T (p.Thr687Ile) 1:181690997 C>T	c.2104G>T (p.Ala702Ser) 1:181693635 G>T	c.2105C>T (p.Ala702Val) 1:181693636 C>T	c.2108T>G (p.Val703Gly) 1: 181693639T>G	c.3422+1G>A (p.?) 1:181705571 G>A
Inheritance	de novo	de novo	de novo	de novo	de novo	de novo	de novo
Localization	S3 of Domain I	Intersegment S1–S2 of Domain II	S6 of Domain II	S6 of Domain II	S6 of Domain II	S6 of Domain II	Beginning of Domain III
Age at genetics assessment	6 yo	8 yo	18 mo	31 yo	6 yo	4 yo 7 mo	6 yo 6 mo
*Clinical characteristics and history*							
Sex	M	M	M	F	M	F	M
Age at onset symptoms/parental concern	14 mo (regression)	8–10 mo	12 mo	6 mo	Shortly after birth	Neonatal GI symptoms related to sucrose isomaltase deficiency; DD at 6 mo	24 mo (regression)
Age at last clinical evaluation	7 yo	10 mo	25 mo	31 yo	6 yo	4 yo	8.5 yo
Seizures	No	No	No	IS at 6.5 mo—ACTH	No	No	No
EEG (age)	Left parieto sagittal discharges (6 yo) activated during sleep	Unremarkable (8 yo)	NA	NA	Multifocal spike and slow wave discharges (4.5 yo); bifrontal sharps activated during sleep	NA	NA
*Neurological evaluation*							
Head circumference at last evaluation (Percentile)	52 cm (P10–25)	51.4 cm (P25–50)	47.6 cm (P10–25)	55 cm (P25–50)	52 cm (P25–50)	48.8 cm (P3–10)	52 cm (P10–25)
Developmental regression number (age)	2 (14 mo, 5 yo) Loss of communication skills, autonomy and diurnal bladder control at 5 yo	1 (4 yo) Fine and global motor skills regression	1 (12–18 mo) Language regression	No	NA	No	1 (24 mo) Language regression
GDD*	+	+	+	+	+	+	+
Intellectual disabilities (> 5yo)**	Moderate	Moderate to severe	NA	Severe	NA	NA	Severe
Language development	Delayed Verbal, able to express complete sentences	Delayed Speaks few words at 8 yo	Delayed No words at 2 yo	Delayed Some single-words	Delayed Non-verbal	Delayed Babbles at 4 yo	Delayed Phrase speech at 48 mo; echolalia
Social behavior/impairment	Abnormal/ASD diagnosis	Abnormal/ ASD diagnosis	None reported at 2 yo	Abnormal	NA	Social: makes eye contact, smiles, giggles	Abnormal/ASD diagnosis
Sensory issues	Produces loud sounds No reaction to pain	Sensory seeking behavior (repetitive tapping)	–	–	Reduced pain sensitivity	Displayed sensory-seeking behaviors	Picky eater Sensitive to noise and touch
Motor stereotypies	Body rocking, hand flapping	Hand flapping	NA	Hand flapping, hand stereotypies	NA	None	Persistent motor stereotypies
Tone disorder	Hypotonia	Hypotonia	Hypotonia	None	Hypotonia	Hypotonia	None
Motor development	Delayed Ambulatory	Delayed Ambulatory	Delayed, Not walking at 2 yo	Delayed Ambulatory	Delayed Not walking, sat at 2.5 yo	Delayed Not walking at 4 yo Apraxia	Delayed Ambulatory

*ACTH* adrenocorticotropic hormone, *DD* developmental delay, *F* female, *GI* gastrointestinal, *IS* infantile spasm, *M* male, *mo* months old, *NA* not available, *S* segment, *yo* year old. *No developmental quotient was available; **For patient 1 a WIPPSI IV was performed; the type of testing used for the IQ assessment was not available for the other patients

The recent articles by Helbig et al*.* [12] suggested the use of the anti-seizure drug topiramate targeting the Cav2.3 channel, encoded by *CACNA1E,* in order to manage the refractory epilepsy present in their patients. After identification of a *CACNA1E* variant in patient 1 in the current study, he received a trial of topiramate on the basis of a hypothesis that even despite the absence of seizures, modulation of intracellular Ca^2+^ influx might positively impact the behavioral phenotype. For this reason, we present a more detailed clinical history of this patient.

Patient 1 presented with marked developmental regression at age of 14 months with loss of first words and communicative gestures. Retrospectively, the development up to that point had been slow, particularly concerning speech development and communication competencies. Subsequently, there was slow progress in language acquisition, and at age 4 years, he was diagnosed with ASD and GDD. At that time, the first regression episode was interpreted as suggestive of an autistic regression.

At age 5 years, he suddenly presented with a second developmental regression with motor hyperactivity, pervasive stereotypies (body rocking and hand stereotypies), loss of interest in play, and loss of sphincter control at night. Brain MRI and a metabolic assessment were normal. EEG showed bilateral occipital spikes, activated during sleep. He never had epileptic seizures. He had a severe sleep disorder. The sleep EEG monitoring excluded nocturnal epileptic seizures as the cause of multiple awakenings. Progressive titration of topiramate up to 2 mg/kg/day was accompanied by improvement in motor hyperkinesia and social contact, and regain of nocturnal sphincter control. A dosage above 2.5 mg/kg/day was not well tolerated because of increased irritability.

### Genetics findings

With exome sequencing, we identified seven novel variants in *CACNA1E* in seven unrelated patients. In individual 1, a de novo missense mutation in *CACNA1E*, c.488T>C, was localized in the segment S3 of Domain I, leading to a p.Met163Thr change. Individual 2 carried a c.1499A>G de novo missense variant in the Domain II of CACNA1E, between segments S1 and S2, leading to a p.Gln500Arg change. Individuals 3 to 6 carried each a de novo missense variant clustering in the segment S6 of the Domain II, c.2060C>T, c.2104G>T, c.2105C>T and c.2108T>G, resulting in p.Thr687Ile, p.Ala702Ser, p.Ala702Val and p.Val703Gly changes, respectively. Individual 7 carried a de novo splicing variant c.3422+1G>A, localized at the beginning of Domain III.

**Fig. 1:**
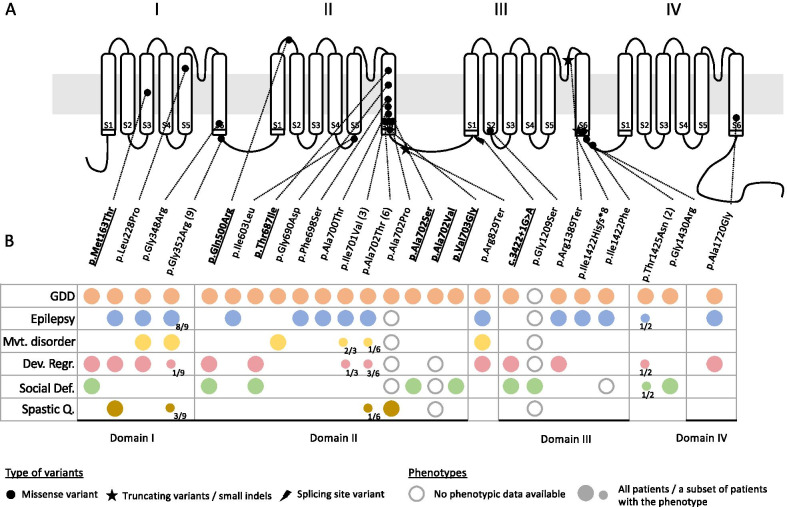
CACNA1E structure and reported variants (**A**) and their impact on the patients’ phenotypes (**B**). Underlined and in bold are the patients described in this study; other patients were described in Helbig et al. [12] and in Heyne et al*.* [11]. The phenotypes include global developmental delay (GDD), epilepsy, movement disorder (Mvt. disorder) including dyskinesia, developmental regression (Dev. Regr.), social deficit (Social Def.) including autism spectrum disorder, hyperactivity, and spastic quadriplegia (Spastic Q.). The lack of circle indicates that the related phenotype is not present in the patient(s) with the variant

These variants’ localizations on the *CACNA1E* structure and domains are shown in Fig. 1A, along with the variants described in Helbig et al. [12] and in Heyne et al*.* [11]. All residues impacted by these missense variants in individuals 1–6 are highly conserved and predicted to be deleterious by various in silico predictors (Additional file 1: Table S3). The splicing variant in individual 7 is predicted by three splicing predictors (MaxEntScan, NNSPLICE, and Human Splicing Finder from Alamut Visual (Interactive Biosoftware)) to disrupt exon 22 consensus donor site. None of the seven novel variants reported here are present in public databases of control individuals (gnomAD [21], LOVD [22]).

*CACNA1E* is predicted to be associated mainly with an autosomal dominant disorder, with a high DOMINO score of 0.995 [23]. Bioinformatic analysis of variant types and distributions in gnomAD control dataset revealed a conspicuous absence of loss-of-function (LOF) variants in *CACNA1E*, suggesting that this gene is highly intolerant to truncating mutations (gnomAD LOF metrics for *CACNA1E*: pLI = 1 and LOEUF = 0.12). *CACNA1E* is also strongly intolerant to missense variation (gnomAD Z score = 5.81) [21].

**Fig. 2:**
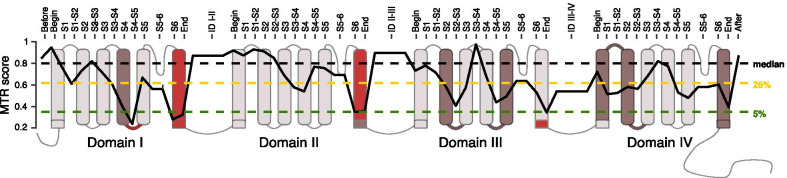
MTR-plot of *CACNA1E*, reflecting intolerance of CACNA1E segments to missense variations. Horizontal dashed lines show *CACNA1E* MTR percentiles 5th (green line), 25th (yellow line) and 50th (black line), and the solid black line indicates mean MTR value of a given CACNA1E segment, intersegment and interdomain. Segments S6 in Domains I and II, intersegment S4-S5 of Domain I as well as ends of Domains I and III, highlighted in dark red color, are considered highly intolerant to missense variation, as their MTR value is below 5th percentile with FDR < 0.1. Regions in darker brown color are considered as being under purifying selection as their mean MTR < 1 and FDR < 0.1 (*S*: segment, D: domain, ID: inter-domain)

We used MTR region-gated analysis of missense variants [19] to infer the functional importance of the different segments and domains of the protein (Fig. 2). The significantly conserved regions (corresponding to MTR score below 0.33) are located in the segment 6 of Domains I and II as well as the regions just at the end of Domains I and III and the intersegment S4-S5 in Domain I (Fig. 2). It correlates with the different clusters of pathogenic variants observed in the reported patients (Fig. 1A).

## Discussion

The seven individuals (six children and one young adult) with heterozygous de novo pathogenic variants in *CACNA1E* described here showed a broad phenotypic spectrum including intellectual disability, GDD, abnormal behavioral phenotypes (in three cases associated with clinically confirmed ASD diagnosis), hypotonia, absence of language or speech delay and developmental regressions in infancy, but no severe neuromotor deficit or epileptic seizures (except for epileptic spasms in Patient 4). This cohort has enabled us to broaden the genotypic and the phenotypic spectrum of *CACNA1E*-related neurodevelopmental disorder.

The phenotype in our patients was milder than the one reported previously [12], which might be linked to the absence of refractory epilepsy. Indeed, the 30 patients previously reported [12] presented with a more severe neurodevelopmental phenotype, as the expression of a more severely impacted brain developmental trajectory. Among them, four had no epilepsy and one had no information available for the epilepsy phenotype. The non-epileptic patients older than 2 years (2/4, 50%) showed a milder phenotype than the others in the cohort, having single-word capacity and being ambulant. Cognitive and behavioral profiles reported for those patients were very similar to the ones seen in our cohort. However, social deficits were not reported in their study.

Pathogenic *CACNA1E* variants reported previously [12] and in this paper cluster mostly inside or at the end of segment 6 of Domains I, II and III (Fig. 1A). Based on the analysis of genetic intolerance, those regions are the most intolerant to missense variation (Fig. 2). Thus, the natural variant epidemiology supports the concept that these regions are most crucial for the normal function of CACNA1E [16, 24].

Based on the clinical description of the patients harboring the different *CACNA1E* variants, there seems to be no clear link between the localization of the variant on the protein and the clinical outcome, especially of refractory epilepsy or spastic quadriplegia (Fig. 1B). Indeed, four of the patients presented here carry missense variants impacting either the same amino acid (p.Ala702) or in the same clustered region of the segment 6 in Domain II as some patients reported with severe epilepsy [12]. Interestingly, among the patients without epilepsy reported previously, two harbor missense variants also seen in patients with severe epilepsy [12]. The first patient is a 1-year-old girl with a p.Gly352Arg missense variant, a recurrent mutation seen in 8 other patients with a strong epilepsy phenotype, which started after age 1 in 3/8 cases. The second non-epileptic patient is a 6-year-old boy carrying a p.Thr1425Asn variant, also seen in a second patient who had epileptic spasms at 5 months of age, but none since then.

Based on the existing reports as well as the case series reported here, pathogenic variants in *CACNA1E* appear to result in a disease with a spectrum of clinical manifestations ranging from severe refractory epilepsy with globally, severely disturbed development (DEE) to GDD with intellectual disability, developmental regression and ASD. This high phenotypic heterogeneity is not uncommon in both adult and developmental channelopathies [25], and it is made even more evident by the observation of different phenotypes resulting from identical mutations. Indeed, it is likely that other mechanisms including modifier genes, environmental factors or variations in gene expressivity contribute to the variability of the phenotypes. Considering the variability in the age of epileptic onset in the cohort of Helbig et al. [12], ranging from 1 day to 3 years, we cannot exclude that the younger patients in our cohort may develop seizures later in life.

The identification of a splicing variant adds further complexity to the pathogenic mechanisms. Our patient 7 has a variant predicted to disrupt exon 22 consensus splicing donor site (c.3422+1G>A). Helbig et al*.* have tested electrophysiologically the functional effects of the three *CACNA1E* missense variants in the segment 6 of Domain II and one variant located in the S4-S5 intersegment of Domain II. These 4 missense variants all led to an increase in current density, facilitated channel activation and slower deactivation, and were thus consistent with gain-of-function effect of the variants. Additionally, the study also described three loss-of-function variants impacting *CACNA1E* and suggested that haploinsufficiency might be another pathogenic mechanism. The phenotypes of those three patients were milder than the others in their study and resemble that of the patients in our series. The presence of both haploinsufficiency variants as well as missense variants that may act through dominant negative or perhaps even gain-of-function mechanism is intriguing, but not novel. The existence of both loss-of-function and gain-of-function variants in calcium channel genes has been reported in *CACNA1A* for variants leading to variable severity of DEE [26], as well as in other neuronal channels (*SCN8A* [27], *KCNQ2* [28], *KCNQ5* [29], *KCNA2* [30], *KCNB1* [31]). Interestingly, gain-of-function changes in the electrophysiology of the cell can also result from loss-of-function effects of variants at the molecular level, and therefore, the gain-of-function effect of truncating and/or splicing variants in *CACNA1E* should not be excluded without further functional studies of these variants. Based on the genetic epidemiology of variants in gnomAD and our computations, the *CACNA1E* gene seems to be constrained both against missense as well as against loss-of-function variants. Thus, the pathogenic mechanism remains unclear and this complicates genotype–phenotype correlation analysis. Such molecular understanding would be therefore needed in the future in order to design therapeutic approaches with drugs such as topiramate.

Furthermore, the observations in this small cohort challenge our current understanding of the interplay between epilepsy and its impact versus its co-occurrence with developmental delay. The term DEE was coined by the most recent classification from the International League Against Epilepsy (ILAE), defining relationship between developmental delay with or without early-life pharmacoresistant epilepsy [32]. However, recent publications of subjects harboring pathogenic variants in so-called epilepsy genes who did not have seizures suggest that the concept of variants specific to epilepsy in the context of syndromic presentation may be mistaken. In that aspect, epilepsy, or ASD, might be considered as one of the phenotypic expressions of a monogenic syndrome [33]. The present work adds *CACNA1E* to the expanding list of genes implicated in an autosomal dominant neurodevelopmental disorder, including ASD, with or without epilepsy. Growing evidence suggests that diagnostic gene panels for epilepsy should be merged with larger brain development gene panels.

In Patient 1, treatment of topiramate [12] was introduced with the hypothesis that modulation of the dysfunctional α1 subunit might improve the function of the neuronal connectivity underlying behavioral disorders, independently of the electroencephalographic activity as the patient never presented with any seizure. Parents reported a qualitative improvement concerning sleep quality and sphincter control; however, it might had happened by chance or as a natural evolution of the disorder. No additional effect has been observed on his behavioral disorder and development.

This study provides a further example of how matchmaking tools like GeneMatcher can be used to define expanded phenotypes associated with described disease genes, like it has been previously described for *LMNB1* [34] and *GATAD2B* [35]. As NGS analysis is becoming routine worldwide and many laboratories are faced with variants that may not correspond to “typical” phenotypes, these sharing and matchmaking tools will become even more important to facilitate the recognition of novel phenotypic associations and variant interpretation.

## Limitations

The main limitation of our study is the absence of functional studies of the effects and impacts of the identified variants on Cav2.3 channel function. Due to this lack of experimental data, we cannot assess whether the variants reported have a gain-of-function effect, as described in Helbig et al. [12]. Considering the similar types of variants (missense) and the consistent NDD phenotype, the de novo origin of these variants, their absence in control population, their impact on conserved amino acid and deleterious predictions and the presence of nearby pathogenic variants, our reported variants are classified as likely pathogenic/pathogenic according to ACMG criteria [18]. Functional experiments are nevertheless essential to understand the pathomechanism behind the reported variants.

The second limitation of our study is the small cohort size. Gathering of patients through matchmaking platforms comes with clear benefits but at the same time is limited by its multi-centric nature, which leads to involvement of variable specialists and assessment tools for each patient. Similarly, the individual submitting the information on the matchmaking platforms might not always be in direct contact with the patients and their referring clinicians, explaining the variability in clinical assessments and detailed history of the patients, for example here the presence/absence of early regression or diagnostic criteria and full assessment for ASD. It thus shows how crucial the collaboration between clinicians and molecular specialists might be in the future.

Topiramate was tested in only one patient of the cohort, and no specific recommendations can be made, as the indication for its use was speculative. However, this information might support the use of this anti-seizure medication drug in unusual indications.

In addition, our study highlights the role of both splicing loss-of-function and missense variants but does not elucidate the underlying molecular mechanisms.

## Conclusion

In this study, we identified heterozygous pathogenic or likely pathogenic de novo variants impacting *CACNA1E* in seven patients presented with a phenotype that was less severe than that previously reported, notably with the absence of severe epilepsy, of macrocephaly and of spastic quadriplegia. The overlapping phenotypes in our patients include GDD, abnormal social behavior including ASD, hypotonia and developmental regression. Furthermore, we analyzed the variant landscape of *CACNA1E* and showed strong constraint of missense variants in specific segments of *CACNA1E*. We conclude that *CACNA1E* variants have a broader phenotypic spectrum than previously reported, including non-epileptic ASD and GDD, and this may inform the composition of diagnostic gene panels and the interpretation of variants observed in NGS studies.

## Supplementary Information

**Additional file 1: Table S1.** Sequencing methods for each patient of the cohort. **Table S2.** Detailed clinical description of CACNA1E patients. **Table 3.** Variants’ description, bioinformatics predictors and ACMG classification.

**Additional file 2.** List of members of the Undiagnosed Diseases Network.

## Abbreviations

ACMG: The American College of Medical Genetics
ASD: Autism spectrum disorder
DEE: Developmental and/or epileptic encephalopathy
FDR: False Discovery Rate
GDD: Global developmental delay
MTR: Missense Tolerance Ratio
NDD: Neurodevelopmental disorder
NGS: Next-Generation Sequencing
VGCC: Voltage-gated calcium channels

## Declarations

### Availability of data and materials

The genetic data supporting the findings of this study, as a whole, contain information that could compromise the privacy and/or consent of the participants. We therefore only provide the causative variants’ details, which are available in the manuscript and tables.
