# Supplementary material for: De novo variants in CACNA1E found in patients with intellectual disability, developmental regression and social cognition deficit but no seizures
Source: Mol Autism. 2021 Oct 26;12:69. doi: 10.1186/s13229-021-00473-3 (PMC8547031; doi:10.1186/s13229-021-00473-3)
Supplement: Supplementary file 2 — Additional file 2. List of members of the Undiagnosed Diseases Network. [file 13229_2021_473_MOESM2_ESM.docx]

**Supplementary note**

**List of members of the Undiagnosed Diseases Network.**

|  |
| --- |
| Maria T. Acosta |
| Margaret Adam |
| David R. Adams |
| Pankaj B. Agrawal |
| Mercedes E. Alejandro |
| Justin Alvey |
| Laura Amendola |
| Ashley Andrews |
| Euan A. Ashley |
| Mahshid S. Azamian |
| Carlos A. Bacino |
| Guney Bademci |
| Eva Baker |
| Ashok Balasubramanyam |
| Dustin Baldridge |
| Jim Bale |
| Michael Bamshad |
| Deborah Barbouth |
| Pinar Bayrak-Toydemir |
| Anita Beck |
| Alan H. Beggs |
| Edward Behrens |
| Gill Bejerano |
| Jimmy Bennet |
| Beverly Berg-Rood |
| Jonathan A. Bernstein |
| Gerard T. Berry |
| Anna Bican |
| Stephanie Bivona |
| Elizabeth Blue |
| John Bohnsack |
| Carsten Bonnenmann |
| Devon Bonner |
| Lorenzo Botto |
| Brenna Boyd |
| Lauren C. Briere |
| Elly Brokamp |
| Gabrielle Brown |
| Elizabeth A. Burke |
| Lindsay C. Burrage |
| Manish J. Butte |
| Peter Byers |
| William E. Byrd |
| John Carey |
| Olveen Carrasquillo |
| Ta Chen Peter Chang |
| Sirisak Chanprasert |
| Hsiao-Tuan Chao |
| Gary D. Clark |
| Terra R. Coakley |
| Laurel A. Cobban |
| Joy D. Cogan |
| Matthew Coggins |
| F. Sessions Cole |
| Heather A. Colley |
| Cynthia M. Cooper |
| Heidi Cope |
| William J. Craigen |
| Andrew B. Crouse |
| Michael Cunningham |
| Precilla D'Souza |
| Hongzheng Dai |
| Surendra Dasari |
| Joie Davis |
| Jyoti G. Dayal |
| Matthew Deardorff |
| Esteban C. Dell'Angelica |
| Shweta U. Dhar |
| Katrina Dipple |
| Daniel Doherty |
| Naghmeh Dorrani |
| Argenia L. Doss |
| Emilie D. Douine |
| David D. Draper |
| Laura Duncan |
| Dawn Earl |
| David J. Eckstein |
| Lisa T. Emrick |
| Christine M. Eng |
| Cecilia Esteves |
| Marni Falk |
| Liliana Fernandez |
| Carlos Ferreira |
| Elizabeth L. Fieg |
| Laurie C. Findley |
| Paul G. Fisher |
| Brent L. Fogel |
| Irman Forghani |
| William A. Gahl |
| Ian Glass |
| Bernadette Gochuico |
| Rena A. Godfrey |
| Katie Golden-Grant |
| Alica M. Goldman |
| Madison P. Goldrich |
| David B. Goldstein |
| Alana Grajewski |
| Catherine A. Groden |
| Irma Gutierrez |
| Sihoun Hahn |
| Rizwan Hamid |
| Neil A. Hanchard |
| Athena Hantzaridis |
| Kelly Hassey |
| Nichole Hayes |
| Frances High |
| Anne Hing |
| Fuki M. Hisama |
| Ingrid A. Holm |
| Jason Hom |
| Martha Horike-Pyne |
| Alden Huang |
| Yong Huang |
| Laryssa Huryn |
| Rosario Isasi |
| Fariha Jamal |
| Gail P. Jarvik |
| Jeffrey Jarvik |
| Suman Jayadev |
| Lefkothea Karaviti |
| Jennifer Kennedy |
| Dana Kiley |
| Shilpa N. Kobren |
| Isaac S. Kohane |
| Jennefer N. Kohler |
| Deborah Krakow |
| Donna M. Krasnewich |
| Elijah Kravets |
| Susan Korrick |
| Mary Koziura |
| Joel B. Krier |
| Seema R. Lalani |
| Byron Lam |
| Christina Lam |
| Grace L. LaMoure |
| Brendan C. Lanpher |
| Ian R. Lanza |
| Lea Latham |
| Kimberly LeBlanc |
| Brendan H. Lee |
| Hane Lee |
| Roy Levitt |
| Richard A. Lewis |
| Sharyn A. Lincoln |
| Pengfei Liu |
| Xue Zhong Liu |
| Nicola Longo |
| Sandra K. Loo |
| Joseph Loscalzo |
| Richard L. Maas |
| John MacDowall |
| Ellen F. Macnamara |
| Calum A. MacRae |
| Valerie V. Maduro |
| Bryan C. Mak |
| May Christine V. Malicdan |
| Laura A. Mamounas |
| Teri A. Manolio |
| Rong Mao |
| Kenneth Maravilla |
| Thomas C. Markello |
| Ronit Marom |
| Gabor Marth |
| Beth A. Martin |
| Martin G. Martin |
| Julian A. Martínez-Agosto |
| Shruti Marwaha |
| Jacob McCauley |
| Allyn McConkie-Rosell |
| Alexa T. McCray |
| Elisabeth McGee |
| Heather Mefford |
| J. Lawrence Merritt |
| Matthew Might |
| Ghayda Mirzaa |
| Eva Morava |
| Paolo M. Moretti |
| Deborah Mosbrook-Davis |
| John J. Mulvihill |
| David R. Murdock |
| Anna Nagy |
| Mariko Nakano-Okuno |
| Avi Nath |
| Stan F. Nelson |
| John H. Newman |
| Sarah K. Nicholas |
| Deborah Nickerson |
| Shirley Nieves-Rodriguez |
| Donna Novacic |
| Devin Oglesbee |
| James P. Orengo |
| Laura Pace |
| Stephen Pak |
| J. Carl Pallais |
| Christina GS. Palmer |
| Jeanette C. Papp |
| Neil H. Parker |
| John A. Phillips III |
| Jennifer E. Posey |
| Lorraine Potocki |
| Bradley Power |
| Barbara N. Pusey |
| Aaron Quinlan |
| Wendy Raskind |
| Archana N. Raja |
| Deepak A. Rao |
| Genecee Renteria |
| Chloe M. Reuter |
| Lynette Rives |
| Amy K. Robertson |
| Lance H. Rodan |
| Jill A. Rosenfeld |
| Natalie Rosenwasser |
| Francis Rossignol |
| Maura Ruzhnikov |
| Ralph Sacco |
| Jacinda B. Sampson |
| Susan L. Samson |
| Mario Saporta |
| C. Ron Scott |
| Judy Schaechter |
| Timothy Schedl |
| Kelly Schoch |
| Daryl A. Scott |
| Vandana Shashi |
| Jimann Shin |
| Rebecca Signer |
| Edwin K. Silverman |
| Janet S. Sinsheimer |
| Kathy Sisco |
| Edward C. Smith |
| Kevin S. Smith |
| Emily Solem |
| Lilianna Solnica-Krezel |
| Ben Solomon |
| Rebecca C. Spillmann |
| Joan M. Stoler |
| Jennifer A. Sullivan |
| Kathleen Sullivan |
| Angela Sun |
| Shirley Sutton |
| David A. Sweetser |
| Virginia Sybert |
| Holly K. Tabor |
| Amelia L. M. Tan |
| Queenie K.-G. Tan |
| Mustafa Tekin |
| Fred Telischi |
| Willa Thorson |
| Audrey Thurm |
| Cynthia J. Tifft |
| Camilo Toro |
| Alyssa A. Tran |
| Brianna M. Tucker |
| Tiina K. Urv |
| Adeline Vanderver |
| Matt Velinder |
| Dave Viskochil |
| Tiphanie P. Vogel |
| Colleen E. Wahl |
| Stephanie Wallace |
| Nicole M. Walley |
| Chris A. Walsh |
| Melissa Walker |
| Jennifer Wambach |
| Jijun Wan |
| Lee-kai Wang |
| Michael F. Wangler |
| Patricia A. Ward |
| Daniel Wegner |
| Mark Wener |
| Tara Wenger |
| Katherine Wesseling Perry |
| Monte Westerfield |
| Matthew T. Wheeler |
| Jordan Whitlock |
| Lynne A. Wolfe |
| Jeremy D. Woods |
| Shinya Yamamoto |
| John Yang |
| Muhammad Yousef |
| Diane B. Zastrow |
| Wadih Zein |
| Chunli Zhao |
| Stephan Zuchner |
